# Cost savings of a primary care program for individuals recently released from prison: a propensity-matched study

**DOI:** 10.1186/s12913-022-07985-5

**Published:** 2022-04-30

**Authors:** Tyler D. Harvey, Susan H. Busch, Hsiu-Ju Lin, Jenerius A. Aminawung, Lisa Puglisi, Shira Shavit, Emily A. Wang

**Affiliations:** 1 SEICHE Center for Health and Justice, Yale School of Medicine, 300 George Street, Suite G05, New Haven, CT 06511 USA; 2 Department of Health Policy and Management, Yale School of Public Health, New Haven, CT USA; 3 School of Social Work, University of Connecticut, Storrs, CT USA; 4 Connecticut Department of Mental Health and Addiction Services, CT Hartford, USA; 5 Department of Internal Medicine, Yale School of Medicine, New Haven, CT USA; 6 Department of Family and Community Medicine, University of California, San Francisco, San Francisco, CA USA

**Keywords:** Medicaid, Criminal justice system, Prison, Costs and cost analysis, Primary care

## Abstract

**Background:**

Criminal justice system costs in the United States have exponentially increased over the last decades, and providing health care to individuals released from incarceration is costly. To better understand how to manage costs to state budgets for those who have been incarcerated, we aimed to assess state-level costs of an enhanced primary care program, Transitions Clinic Network (TCN), for chronically-ill and older individuals recently released from prison.

**Methods:**

We linked administrative data from Connecticut Department of Correction, Medicaid, and Department of Mental Health and Addiction Services to identify a propensity matched comparison group and estimate costs of a primary care program serving chronically-ill and older individuals released from incarceration between 2013 and 2016. We matched 94 people released from incarceration who received care at a TCN program to 94 people released from incarceration who did not receive care at TCN program on numerous characteristics. People eligible for TCN program participation were released from incarceration within the prior 6 months and had a chronic health condition or were over the age of 50. We estimated 1) costs associated with the TCN program and 2) costs accrued by Medicaid and the criminal justice system. We evaluated associations between program participation and Medicaid and criminal justice system costs over a 12-month period using bivariate analyses with nonparametric bootstrapping method.

**Results:**

The 12-month TCN program operating cost was estimated at $54,394 ($146 per participant per month). Average monthly Medicaid costs per participant were not statistically different between the TCN ($1737 ± $3449) and comparison ($1356 ± $2530) groups. Average monthly criminal justice system costs per participant were significantly lower among TCN group ($733 ± $1130) compared with the matched group ($1276 ± $1738, *p* < 0.05). We estimate every dollar invested in the TCN program yielded a 12-month return of $2.55 to the state.

**Conclusions:**

Medicaid investments in an enhanced primary care program for individuals returning from incarceration are cost neutral and positively impact state budgets by reducing criminal justice system costs.

**Supplementary Information:**

The online version contains supplementary material available at 10.1186/s12913-022-07985-5.

## Background

The cost of the criminal justice system in the United States (US) continues to grow exponentially [1, 2]. The annual cost of operating public prisons and jails, parole, and probation is estimated at $81 billion [3] and exceeds $181 billion dollars when including direct operations, such as the cost of policing and the court system, as well as costs paid by families to support incarcerated family members [2]. Notably, health care is a large driver of correctional system costs. The nation’s correctional system has increasingly housed a larger population of people with substance use disorders and co-morbid conditions, including HIV and hepatitis C, and mental health disorders, which are costly to the system [4, 5]. A systematic review found that among the top 20 countries in terms of prison population, only 10 reported prison healthcare expenditure data, and the US reported spending more of its correctional budget on health care than 8 of the other countries [6]. Delivering care within correctional facilities is complex—not only do incarcerated individuals have high medical needs, [7] but also receipt of care requires involvement of correctional staff for within facility and off-site transportation to receive health care services, which adds to cost.

Largely unaccounted for in these estimates are the costs incurred once people are released from incarceration back to the community, especially use of the community healthcare system. People with histories of incarceration have high rates of chronic medical, mental health (i.e., schizophrenia, post-traumatic stress disorder), and substance use disorders, which tend to be costly health conditions [5, 8]. Many of these health conditions are inadequately treated during incarceration [9]. Those recently released from incarceration generally experience worsening of chronic health conditions and have disproportionately high rates of emergency department (ED) use and hospitalizations [10, 11]. A 2014 study estimated the healthcare costs for people with past year criminal justice system involvement included an additional $8.5 billion in hospital expenditures and $5.2 billion in ED expenditures, which are largely borne by state Medicaid programs [11].

Given the outsized healthcare costs, state governments are looking to identify and scale programs that improve patient health and reduce the reliance on the criminal justice system for individuals with mental health and substance use disorders, while minimizing overall costs [12]. Further, there is bipartisan support to extend the Medicaid Reentry Act would provide Medicaid coverage for incarcerated individuals for 30 days prior to their release to improve while Medicaid currently does not pay for health services for the duration of individual’s incarceration, coordination and continuity of care between correctional and community health systems prior to release through the Medicaid Reentry Act. Such coverage would have potentially large effects on health and health care costs as individuals reenter their communities [13]. However, few health system interventions targeting individuals following release from incarceration have been studied, and most have not been subjected to economic analysis, which suggests it is unknown if such interventions provide potential cost savings when considering health system and correctional systems costs [14–19].

We present a cost analysis of the Transitions Clinic Network (TCN) program, the largest national network of primary care programs that addresses the health and social needs of people recently released from correctional systems [17, 20–24]. Previously, TCN programs have been shown to be associated with a reduced likelihood of an ED visit [20]. In other work, those who received TCN care were less likely to be reincarcerated for a parole or probation technical violation and had fewer reincarceration days following release from incarceration compared to a comparison group who did not receive TCN services. Similar, among those hospitalized in the 12-month period following release, TCN participation was associated with a reduced length of hospital stay and decreased rates of preventable hospitalizations [21].

This current work adds to our knowledge in its focus on the costs of TCN. We use data from a past quasi-experimental study of the TCN program in Connecticut to estimate health care and criminal justice costs associated with program participation and calculate a return on investment of the program [21]. Documenting the costs of TCN programs is critical in that organizations considering adopting this model may use this information in decision making and budgeting, which will advance programmatic and operational decisions of the network as a whole, but also whether and how investments are made in such programs for this population. This analysis provides important insights for state policymakers on whether investments in Medicaid to support enhanced primary care programs during the immediate period following release (or even prior to release, as could be facilitated by the Medicaid Reentry Act) offers financial benefits for the state. Lastly, it adds to a relatively limited evidence base of the cost of healthcare interventions for individuals leaving incarceration and meeting the needs of people experiencing marginalization [25–27].

## Methods

### Setting

The TCN is a national consortium of 45 primary care based programs that serve the health needs of individuals returning to the community from incarceration in 14 states and Puerto Rico [17]. Each program is based in an existing community health center and focuses on providing enhanced primary care to people released from correctional facilities who have a chronic health condition or who are older than 50 years of age. Participation and engagement with TCN are completely voluntary. Individuals are referred to a program by correctional systems prior to release from incarceration, from community service providers, identified through outreach conducted by TCN community health workers, or self-referral. Interdisciplinary teams, consisting of primary care providers and formerly-incarcerated, specially-trained community health workers, work with patients to address a myriad of health conditions, including substance use disorders and mental health conditions, which may underpin past incarceration history. The team also attends to social determinants of health related to their return from incarceration, such as housing, food access, or employment, and link patients with community agencies. The community health workers use their personal experience of incarceration to educate the healthcare team about patients’ challenges, facilitate patient–provider communication, and help patients navigate and build trust in the medical system. Further, the TCN community health workers and care teams build relationships with public defenders and probation and parole officers and can add medical context to situations which might otherwise lead to reincarceration, such as relapse to substance use or poorly controlled mental health.

Connecticut is a unique state to study costs of healthcare interventions targeting people just released from corrections. It is one of six states with a unified prison-jail system where jails and prisons are under the authority of the state, which provides the opportunity to ascertain costs related to both prison and jail incarceration. Connecticut’s Medicaid program is one of four with a fee-for-service model, unlike most states which have transitioned to a managed care model [28–30]. The state’s Medicaid program covers the costs of health services and visits received through a TCN program but does not cover additional TCN program costs, including the salary of the community health workers based in primary care clinics, supervision costs, nor equipment and transportation needs of the community health worker. Medicaid beneficiaries mostly receive substance use and mental health treatment and support services through health systems that are billed to Medicaid. A small fraction of such services is provided through the state’s Department of Mental Health and Addiction Services, which includes state mental health hospitals and sober houses.

### Study population

We used data from a previous study to assess the impact of the TCN program in New Haven, CT, on the state’s Medicaid and criminal justice system costs [21]. These data were collected as part of a larger, multisite grant funded by the Center for Medicare & Medicaid Innovation during a time in which Medicaid was being expanded under the Affordable Care Act. As described previously, 94 TCN patients seen within 6 months of their prison release who had at least one chronic health condition or were over the age of 50 years old were matched with a comparison group. To create a similar comparison group, we identified 2594 individuals who were released from prison to another medium-sized city in Connecticut that did not offer TCN care at the time of the study. Using linked administrative data from Connecticut Department of Correction, Connecticut Medicaid, and the Connecticut Department of Mental Health and Addiction Services and detailed participants’ information, we estimated propensity scores and then utilized a greedy matching algorithm without replacement to create a 1:1 case-control sample [21]. We used 32 covariates to estimate the propensity scores based on all available information in the linked administrative data in the following domains: demographic variables, criminal justice system history, Department of Correction assessment scores, and medical and mental health history. After matching, participant demographics, severity of past criminal justice history, chronic medical conditions, mental health conditions, substance use treatment, and previous healthcare use were not different between the TCN and matched comparison groups (Supplement, Table 1). Additional information on our propensity score matching methodology is provided in the Supplement.

### Conceptual framework

**Fig. 1:**
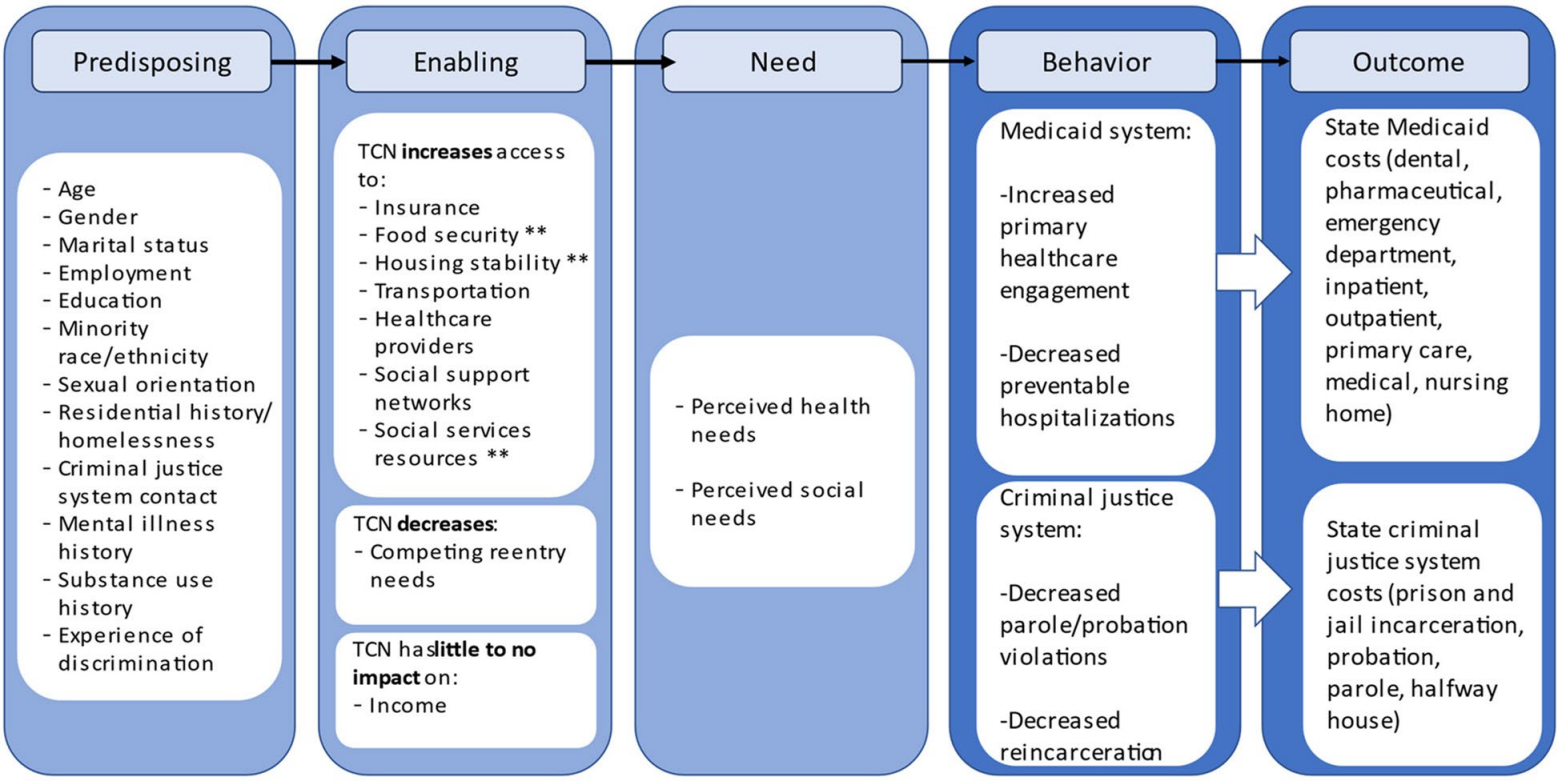
Applying the Gelberg-Anderson Behavioral Model for Understanding Transitions Clinic Network Intervention’s Potential Impact on State Medicaid and Criminal Justice Costs. ** Represent costs that were unmeasured in this study due to data limitations but predicted to have potential impacts on state budgets

To guide our theoretical framework for how the TCN program may affect state Medicaid and criminal justice costs, we use the Gelberg-Anderson Behavioral Model for Vulnerable Populations to understand potential costs to the state among individuals released from correctional systems (Fig. 1) [31]. Within this framework, TCN programs act to modify the majority of enabling factors leading to state Medicaid and criminal justice costs, such as increasing access to housing stability through TCN CHWs helping patients with housing and other public social service (i.e., food) programs. One plausible way TCN engagement could impact costs is by anticipating health and social needs so that the patient does not end up needing costly acute care; however, treatment of some highly prevalent conditions, including hepatitis C, may introduce higher costs even in the ambulatory care setting. In fact, based on past studies, TCN programs shift health care use from acute care settings to primary care by increasing primary care engagement and decreasing preventable hospitalizations and reduce criminal justice contact through decreased parole and probation violations as well as less reincarceration experiences. TCN programs also make appropriate referrals to substance use and mental health treatment, as well as advocate on patients’ behalf in interactions with the criminal justice system, especially courts, probation and parole when appropriate. In these ways, TCN programs may avoid some costly health services, including acute care services but also gain access to other health services they would have not (i.e., hepatitis C treatment) and criminal justice system costs.

### Measures

We assigned each study participant an index date: admission date for TCN program and date of release from the correctional system for the comparison group. We then calculated costs over a 12-month period following each participant’s index date using data from the Connecticut Department of Correction and Medicaid. We used Medicaid claims data to calculate total 12- month follow-up period cost per participant. Costs were categorized using preset Medicaid categories including dental, pharmaceutical, ED, inpatient, outpatient (i.e., outpatient hospital), primary care (i.e., services and visits provided by primary care providers), medical (i.e., physician services and other medical costs, including radiology), nursing home, and crossover (i.e., claims for dual-eligible Medicaid/Medicare beneficiaries). TCN participants and the comparison group were covered by Medicaid for the duration of the study period, unless participants were re-incarcerated. Medicaid costs per participant for each arm was defined as total paid amount from Medicaid claims in the 12 months following the index date. We did not have access to Connecticut Department of Mental Health and Addiction Services payment records and were unable to calculate the costs to this department or other state services including food stamps or housing in our analyses.

To measure costs to Connecticut’s criminal justice system, we obtained facility-specific per diem costs of prison and jail incarceration, costs per day on probation from the Court Support Services Division, and costs per day in a halfway house or on parole from the Department of Correction (FY 2013–2016). The costs per diem while incarcerated ranged from $119 to $512 (based on facility), and the costs per diem on parole and probation were $16 and $12, respectively. Costs per diem incarcerated in jail or prison include health care costs, while costs per day on parole and probation do not, and these health care costs continued to be paid by Medicaid.

We calculated the TCN program cost by summing annual salary and fringe benefit support for a community health worker employed at a federally qualified health center, salary and fringe benefit support for supervision of the community health worker, equipment (cell phone, data plan, laptop computer, office space) and transportation costs. These costs are not covered by state Medicaid or the criminal justice system and were only incurred by the TCN program. We estimated the cost of the TCN program per participant by dividing total annual TCN costs by the approximate number of participants one community health worker would manage in each year during the study period (2013–2016). We assumed approximately 31 patients per 1 community health worker per year given that 94 patients were enrolled in the TCN program over a time frame of 3 years (2013–2016) and that this patient load is standard for CHWs [32].

To calculate the potential cost savings of the TCN program, we calculated the total 12-month Medicaid and criminal justice system costs for both the TCN and comparison groups. We then subtracted these total costs of the TCN group from the comparison total costs to represent the savings to the state realized by the TCN program. The return on investment was realized by dividing this savings by the total annual TCN program costs. Because Medicaid is a state-federal partnership with the federal government covering some of Medicaid costs, the return on investment we calculate is likely an underestimate of any return on investment to state. Importantly, Connecticut has the minimum Federal Medical Assistance Percentage at 50%, which is a percentage that varies across states based on the mean per capita resident income. All costs were adjusted to 2015 dollars using the Consumer Price Index.

### Statistical analysis

We compared participants in each group following propensity score matching on sociodemographics, chronic conditions, and criminal justice and mental health history using t-tests and chi-square tests. To evaluate the associations between the TCN program with costs to the state Medicaid and criminal justice systems, we used t-tests to examine each group’s’ per participant per month differences in costs. To avoid making unrealistic assumptions regarding the distribution of the costs for the underlying population, we performed nonparametric bootstrapping method that applied 1000 replicated random sampling with replacement for the TCN group and the propensity-matched comparison group [33]. For any analyses where assumptions were not met, we report the mean differences based on adjusted degree of freedom for equal variances not assumed. As a sensitivity analysis, we evaluated the associations between TCN participation and costs excluding individuals dually-eligible for Medicaid-Medicare to evaluate if this population skewed any associations. Last, as a post-hoc sensitivity analysis, given the difference in index dates (i.e., admission date for TCN program and date of release from the correctional system for the comparison group) in potentially exacerbating costs, we made an assumption that all TCN cases with probation started incurring probation costs on the day of their release from incarceration. We then added the time frame from release to TCN enrollment to the number of probation days for the TCN participant and compared costs to ensure findings were not skewed. We considered *p*-values of equal to or less than 0.05 statistically significant. All statistical analyses were performed using SAS version 9.4 (SAS Institute Inc., Cary, NC). TCN program participants in New Haven provided informed consent for participation in this study and the linkage of their data to administrative databases. The institutional review boards of Yale University School of Medicine, Connecticut Department of Mental Health and Addiction Services, and the US Office of Human Research Protections approved this study. All research was conducted in accordance with the Declaration of Helsinki.

## Results

The total 12-month cost of the TCN program in Connecticut was estimated to be $54,027, which was driven largely by salary support and fringe for the community health worker and supervising social worker. The estimated cost per participant per month was $146, assuming a standard patient load of approximately 31 patients per year for the community health worker (Table 1) [32]. The mean age of TCN participants was 42.6 (standard deviation:10.4 years). Eighty percent of TCN participants were male, and 54.3% were Black people. The majority of TCN participants had a chronic health condition; 20.2% had diabetes; 38.3% were diagnosed with opioid use disorder, and 53.2% with alcohol use disorder (Supplement, Table 1). The average amount of time between release from incarceration to TCN enrollment for our sample was 47.4 days (±44.9 days), and the median was 28 (range 1–187) days.

**Table 1:** Annual costs associated with a transitions clinic network program in connecticut

	Quantity	Unit Price	Annual Expenditure	Description
**Human Resources**				
Community Health Worker	1 full-time equivalent	$18/h	$38,441^a,b^	Salary support for a trained community health worker who has personally experienced incarceration.
Supervision (Social Worker)	0.1 full-time equivalent	$25/h	$10,552^b^	A supervisor typically meets weekly with community health worker to prepare for clinical visits, works with community health worker during patient visits, and acts as a resource to address patient concerns.
**Office Space and Equipment**				
Office Space Rent	8 square feet/month	$12/square foot	$1178	Office space rental and supplies for community health worker, typically housed within a federally-qualified health center.
Desk and Chair	1^c^	$544	$544	
Laptop Computer	1	$1450	$1450	
Docking Station	1	$226	$226	
Monitor	1	$204	$204	
Key Board, Mouse Pad	1	$45	$45	
**Communications**				
Cell Phone	1	$362	$362	A cell phone and plan allow community health workers to remain in close contact with patients, including scheduling primary care visits, addressing concerns, and assisting with access to other social services.
Data Plan	1	$63/month	$761	
**Transportation**	100 miles/month	$0.5/month	$631	Transportation cost include community health worker and patient travel, clinical visits, patient transportation, and accessing supplies for clinic and patients.
**Total Annual Program Cost**			$54,394	
**Annual Program Cost Per Participant**^d^			$1755	

^a^ This salary aligns with the median reported wages for community health workers in Connecticut in 2015 [34]

^b^ Includes fringe

^c^ 1 indicates one time purchase necessary for supporting a newly hired community health worker

^d^ We assumed 31 patients per 1 community health worker per year given that 94 patients were enrolled in the Transitions Clinic Network program over a time frame of three years (2013–2016)

**Fig. 2:**
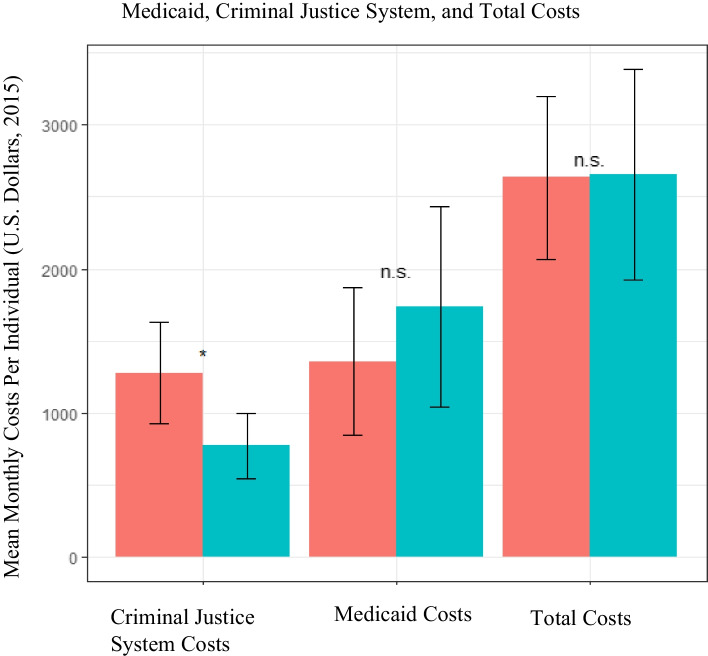
Average Monthly Medicaid, Criminal Justice System, and Total Costs By Group. Average monthly Medicaid, criminal justice system (CJS), and total costs per participant in the Transitions Clinic Network (TCN) versus the matched comparison group with 95% confidence intervals. * indicates significance at the 0.05 level while n.s. indicates no significant different

The average monthly cost, inclusive of Medicaid and criminal justice costs as well as the TCN program costs that cannot be charged to Medicaid for the TCN group, was $2656 (± $3604) per participant for the TCN group, compared to the comparison group of $2633 (± $2788) per participant (Fig. 2). The average monthly Medicaid costs per participant was $1737 in the TCN group compared to $1356 for the comparison group. Within Medicaid costs, average monthly pharmaceutical costs ($528 ± $2244 per participant in the TCN group versus $315 ± $952 per participant in the comparison group), average monthly inpatient costs (TCN, $563 ± $2152 per participant versus comparison, $294 ± $1138 per participant), and average monthly primary care costs (TCN, $324 ± $813 per participant versus comparison, $381 ± $1422 per participant) were not statistically different between the two groups (Table 2).

**Table 2:** Average monthly medicaid costs per individual in transitions clinic network and matched comparison groups^a^

	Transitions Clinic Network (*n* = 94)		Comparison Group (*n* = 94)		Mean in Differences (95% confidence interval)
	Mean	Standard Deviation	Mean	Standard Deviation	
Dental	$34	$52	$31	$54	3 (−13,18)
Pharmaceutical	$528	$2243	$315	$952	213 (−215,726)
Emergency Department	$51	$104	$44	$71	6 (−17,35)
Inpatient	$563	$2152	$294	$1138	269 (−178,788)
Medical^b^	$227	$291	$181	$278	46(−30,126)
Nursing home	$0	$4	$28	$273	−28(−94,1)
Outpatient^c^	$10	$43	$80	$414	−70(− 158,-3)
Primary care^d^	$324	$813	$381	$1422	−58(−394,245)
Crossover^e^	$1	$5	$2	$13	−1(−5,1)
**Total Medicaid**	$1737	$3449	$1356	$2530	381(−470,1259)

^a^ Significance is based on bootstrap results of 1000 samples. No significant differences were detected between the Transitions Clinic Network and matched comparison group

^b^ The medical category include physician services and other medical costs, including radiology

^c^ Outpatient refers to services received in outpatient hospital setting

^d^ Primary care includes services and visits provided by primary care providers

^e^ Crossover includes claims for dual-eligible Medicaid/Medicare beneficiaries

The average monthly criminal justice system cost per participant in the TCN group was $773 (± $1130) and for the comparison group, $1276 (± $1738) (*p* < 0.05; Fig. 1). The average monthly incarceration costs were not statistically different between the two groups, with the TCN group at $539 (± $1064) per participant and the comparison group at $791 (± $1663) per participant (Table 3). Average monthly probation costs were significantly lower among the TCN participants ($33 vs $112 per participant, *p* < 0.001). The average monthly parole costs (TCN group $58 ± $178 per participant versus comparison $90 ± $264 per participant) and halfway house costs (TCN group $142 ± $470 per participant versus comparison $282 ± $697 per participant) were not statistically different between the two groups (Table 3).

**Table 3:** Average monthly criminal justice system costs per individual in transitions clinic network and matched comparison groups

	Transitions Clinic Network (*n* = 94)		Comparison Group (*n* = 94)		Mean in Differences (95% confidence interval)	Significance^a^
	Mean	Standard Deviation	Mean	Standard Deviation		
Jail/Prison	$ 539	$1064	$791	$1663	− 252(−633,165)	–
Probation	$34	$83	$112	$166	−79(− 116, −42)	< 0.000
Halfway housing^b^	$ 142	$470	$282	$697	− 140(− 312,35)	–
Parole	$58	$178	$90	$264	−32(−99,32)	–
**Total Criminal Justice System Costs**	$773	$1130	$1276	$1738	−503 (− 911,-96)	< 0.05

^a^ Significance is based on bootstrap results of 1000 samples

^b^ Halfway housing refers to various residential facilities in which individuals are released from incarceration to live in prior to reentering their communities

In total, the 12-month combined Medicaid and criminal justice costs for the entire TCN group was $2,831,602, while the total 12-month costs for the comparison group was $2,969,503. The TCN intervention thus resulted in a cost reduction of $137,902, or a 5% reduction. This difference divided by the cost of the TCN program of $54,394 yielded a return of $2.55 per dollar invested in the TCN program.

Our sensitivity analysis showed that excluding dually-eligible Medicare-Medicaid participants produced the same results with the criminal justice system costs remaining significantly lower among the TCN group ($753 ± $1109 per TCN participant versus $1307 ± $1757 per participant in comparison group) (Table 2, Supplement). Our additional sensitivity analysis showed that even after the inclusion of those additional probation costs to the TCN group, the TCN group still has significant lower probation than the comparison group (TCN: $40 ± $96 per TCN participant per month versus $112 ± $166 per participant in comparison group per month; *p* < 0.001) (Table 3, Supplement).

## Discussion

Within a 12-month period, a TCN program yielded an annual return of $2.55 for every dollar invested, when considering Medicaid and criminal justice system costs. This return on investment was primarily driven by differences in monthly criminal justice system costs per participant, not Medicaid costs, between the two groups, as TCN participants had reduced probation costs. This result confirms our previous work showing that participation in TCN lowered the odds of reincarceration due to a parole or probation technical violation and confirms there are cost benefits to such an effect. Interestingly, we did not find significant differences in Medicaid costs between the group, despite our previous findings that TCN participation shortens hospital stays and lowers preventable hospitalizations for individuals hospitalized [20]. This could be a function of our sample size.

While our analysis does not establish the mechanism by which TCN lowers probation costs, there are plausible mechanisms to explain the association found. First, TCN patients may better be able to address their health and social needs, allowing them to meet probation requirements, which is consistent with other intervention work focused on this population [35]. Another possible reason is that TCN program providers and community health workers communicate regularly (when given permission by their patients) with criminal justice entities, especially parole and probation officers. These relationships may be important in identifying alternatives to re-incarceration for technical violations, including enrolling patients in substance use treatment when individuals have relapsed to drug use. Such a potential mechanism emphasizes the importance of the TCN intervention being embedded within the healthcare system to have the expertise and resources to fully understand a person’s health context.

To our knowledge, this is the first economic analysis of a US health system–based community health worker intervention for adults leaving the correctional system. Our analysis provides a more thorough estimate of the return on investment compared with estimates derived from a single source (either Medicaid or the criminal justice system) or pre-post trial designs, and further advances the evidence on how to best meet the needs of individuals belonging to a highly marginalized population. To be sure, other costs to the state were unmeasured, including housing, employment, and food access programs, and reflect future work that can better delineate how health system investments may affect state costs for this population. These data add to the limited evidence examining costs of healthcare interventions for individuals released from incarceration on criminal justice expenditures [25–27]. In contrast to our findings, a study of 1325 recently released individuals in Australia found that individuals randomized to a low-intensity case management program (the ‘Passports Study’) had higher health, criminal justice, and intervention costs, with an average increase of $1790 AUD per participant over 2 years compared to a control group [36]. The key difference in our study and the ‘Passports Study’ is that the TCN intervention has been found to have no impact on preventable ED visits in Connecticut, while the ‘Passports Study’ led to more healthcare utilization, including ED visits, which are costly [36, 37].

Policy makers and criminal justice and health care organizations interested in making similar investments utilizing Medicaid dollars to impact criminal justice expenditures should interpret this study in the context of four key points. First, the financial value of a TCN program depends on the baseline costs among the targeted patient pool. Given that individuals leaving incarceration generally have high health needs, there is a limited need to target the interventions to contain a high-risk patient pool. The program is offered to those in need of primary care with a chronic health condition and those over 50 years of age, which is a broad and medically complex group. Even when participation includes a broader population, and not focused on a specific disease category or a predetermined “high cost group,” the community health worker intervention returned $2.55 for every dollar invested.

Second, return on investment relies critically on who is making the investment and who is receiving the return. We have presented an economic analysis from the perspective of a state, especially as it is responsible for both Medicaid and the correctional system. It is especially important to note that our findings may not generalize to other states given the unified prison-jail system and Medicaid fee-for-service model in Connecticut, nor does it account for the costs of Medicaid to the federal government. We found that the TCN program was cost neutral for Medicaid (i.e., was not significantly different than care as usual), but we would predict that such findings may be different in systems with Medicaid managed care which may incentivize prevention and value-based care.

Third, this study suggests that TCN programs are beneficial, even from a narrow financial perspective. That said, the financial return on investment underestimates the true societal return because the benefits of the program related to improvements in health, remaining in the community, employment and labor market outcomes, or even spillover effects to families and communities are not assessed. For example, some interventions such as recommended cancer screening or identifying patients with chronic conditions like hypertension through community outreach may not affect healthcare costs over a 12-month time horizon, but may lead to valuable improvements in health (and lower costs, over time) [38].

Last, returns that accrue to the state from investing in TCN programs are important, especially as states have long desired to control criminal justice system and health care costs and have worked to reduce incarcerated populations before and during the COVID-19 pandemic. Before COVID-19, the Pew Center on the States surveyed 41 states and estimated that if these states reduced their recidivism rates by 10%, 635 million dollars would be saved in 1 year [39]. In the context of COVID-19, as states across the US release people from correctional facilities in order to mitigate virus transmission, there is a dire need to ensure those individuals access high quality, effective primary care and do not return to the correctional system, which would lower both healthcare and criminal justice system costs [12]. Criminal justice reforms and decarceration efforts that address the unique health needs of this population may both address the health-harming effects of incarceration, but also minimize additional costs to states.

### Limitations

Our findings may not generalize to other states given the Medicaid fee-for-service model in Connecticut. The relatively short 12-month, follow-up period may underestimate the health- related costs associated with medical treatment if its effects of such treatment persist over the longer run, like hepatitis C treatment. Alternatively, the additional benefits accrued beyond the 12-month window might be offset by additional costs if patients require further medical treatment. Next, we chose an index date of TCN enrollment for the TCN group and release from incarceration for the comparison group, which may introduce bias if participants in the TCN group were likely to engage in costlier care prior to enrollment in the TCN program and following their release from incarceration. That said, we found that the mean number of days for TCN participants in this sample to enroll in the program was approximately 48 days following release from incarceration, and that the overwhelming majority of individuals in the comparison group (85%) did not have any ED visits, hospitalizations, or reincarceration experiences during the first 48 days following their release from incarceration—suggesting this concern did not likely influence our findings [21]. Thus, given the index date for TCN group was admission to the program and the comparison group was release from incarceration, it is still plausible that the difference in index date accounts for a difference in the observed costs though our sensitivity analysis suggests that this is unlikely. While we selected a comparison group from a similar urban area as New Haven, regional differences, such as differences in availability of reentry programs or judges’ behaviors, between the two cities may play a role in our findings. While case law of Connecticut applies to cities across the state uniformly, probation practices might be different in New Haven and the comparison community, leading to different rates of probation violations. We used propensity score matching to create a one-to-one comparison of individuals in the TCN and comparison groups, and while we were able to appropriately balance the groups on numerous sociodemographic and health characteristics, it should be noted that propensity score matching has limitations, including an inability to measure and account for unobservable covariates [40, 41]. Because TCN engagement was completely voluntary, individuals who participated may have a different level of health-seeking behaviors than other individuals leaving incarceration and that difference is not captured by the propensity match. A randomized trial would be best for establishing the relationship between TCN participation and reduced probation costs, particularly randomization at the individual-level, rather than the site, in order to better understand whether effects remain across counties. Our sample size was limited to 94 TCN patients, which impacted our confidence intervals and limits our ability for conclusions that this sample is generalizable to the formerly incarcerated population with chronic health conditions. We were unable to access the cost of services provided through the Department of Mental Health and Addiction Services, which could be potentially expensive, and our results should be interpreted in light of this limitation given that participants in both the TCN and comparison groups could have utilized such services. Our data is from 2013 to 2017, and costs may have shifted since then, but we adjusted our data for inflation costs to 2015.

### Policy implications

Our study suggests that investment in enhanced primary care may be advantageous to state budgets as they attempt to manage the rising costs of correctional systems, while providing evidence-based care to individuals released from incarceration [17, 20, 21]. Policies that provide funding to support community health centers in implementing a TCN program through hiring formerly incarcerated community health workers into healthcare teams could be beneficial by decreasing criminal justice costs and improving health outcomes among individuals released from incarceration. Extending Medicaid funding to compensate for the community health worker’s time and promoting enhanced care management programs that target individuals recently released from incarceration could incentivize adaptation of TCN programs in community health centers. Lastly, this study has demonstrated the benefit of studying the costs of primary care-based programs beyond the health care system. Our findings were dependent on our ability to link Medicaid claims to criminal justice system costs, which is typically unfeasible. The inability to link such data may lead to biased estimates of interventions for individuals recently released from incarceration. States should invest in data linkage systems that facilitate cost analysis across even more systems (i.e. federal criminal justice system, social service agencies) to allow for quantifying benefits of intervention programs from a larger societal perspective. This would enable studying the collateral benefits (i.e. employment, food access) and changes to both individual and family well-being.

### Future research

Future studies exploring the long-term cost impacts of primary care-based programs targeted for people released from corrections are critical to health system and criminal justice system reforms. More attention should be given to investigating the mechanisms by which programs impact costs, such as how TCN participation reduced probation costs in this study. Future analyses should consider benefits specific to certain health conditions and treatment options, including but not limited to substance use disorder treatment or preventative treatment such as cancer screening. These studies will provide evidence for how to mitigate the high costs of the criminal justice system and health systems.

## Conclusion

We find that state investment in TCN programs, an enhanced primary care for individuals recently released from correctional systems, may reduce criminal justice costs, especially through decreased interactions with probation. State policies that fund community health centers to implement such a program for individuals released from incarceration could facilitate cost savings.

## Supplementary Information

**Additional file 1.**

## Data Availability

Original de-identified data can be requested after permissions are obtained from the Connecticut Department of Correction, the Department of Mental Health and Addiction Services, and the Department of Public Health.

## Abbreviations

ED: Emergency department
FY: Fiscal year
HIV: Human immunodeficiency virus
TCN: Transitions Clinic Network
US: United States
