# Supplementary material for: Cost savings of a primary care program for individuals recently released from prison: a propensity-matched study
Source: BMC Health Serv Res. 2022 Apr 30;22:585. doi: 10.1186/s12913-022-07985-5 (PMC9059905; doi:10.1186/s12913-022-07985-5)
Supplement: Supplementary file 1 — Additional file 1. [file 12913_2022_7985_MOESM1_ESM.docx]

**Supplement**

**Methods: Propensity Score Matching**

***Selection of Covariates and Estimation of Propensity Scores***

We included 32 covariates in the estimation of our propensity scores, based on whether the measured variables are related to treatment assignment, or were confounders for outcomes. Those covariates came from the linked administrative dataset and encompass four major domains*:* gender, race/ethnicity and age at release, 2) criminal justice involvement history: release year, Department of Corrections medical, mental health, substance use and criminal justice needs assessment scores (including violence, sentence length, discipline record and gang involvement), criminal justice involvement history (including having a felony record, the percentage of one’s life interacting with the Department of Corrections), type of release (under supervision or end of sentence), 3) behavioral health history: mental health and substance abuse diagnosis and treatment history, including proportion of one’s life as a client of Department of Mental Health and Addiction Services, 4) medical history: having any chronic condition, total number of chronic conditions, having 1 of 17 medical conditions included in the Charlson Comorbidity Index, the weighted Charlson Comorbidity Index score, and three common chronic conditions among individuals with a history of incarceration, but not otherwise included in the comorbidity index score: other gastrointestinal symptoms, opioid use disorder and alcohol use disorder. We utilized logistic regression to estimate the propensity scores.

***Comparison of Propensity Score Models***

To ensure the selection of the most appropriate propensity score method, we compared the use of these different propensity scores models in case-control matching, quantile stratification, and inverse probability weighting (IPW) in terms of the overlapping in the propensity scores by generating boxplots of all estimated propensity scores for each the TCN and comparison groups, and we visually inspected to ensure overlap. We also utilized standardized mean differences to evaluate these different propensity scored models by extent to which they balanced covariables. Based on the analyses, we selected the method which created the most balance of covariates between the TCN and comparison groups. The 1:1 greedy matching algorithm, in which TCN cases were ordered based on propensity score and sequentially matched without replacement to its nearest non-TCN individual, yielded the least biased and most balanced sample. The standardized mean differences show all covariates were balanced between the TCN and matched comparison group (Supplement, Table 1). Due to the extremely unequal sample sizes between the two groups prior to matching (TCN: n=95 versus comparison: n= 2594), quantile stratification failed to produce sufficient overlaps in covariate balance in all strata. Similarly, the extreme unequal sample size also leads to skewed weight distribution (close to 0) for the inverse probability weighting, and the adjusted standardized differences between TCN and comparison group show a less balance model comparing to the 1:1 greedy matched model, with 3 covariates unbalanced, and 7 covariates show increase in the differences between the study group.

**Tables**

| **Table 1. Participant Characteristics in Transitions Clinic Network and Matched Comparison Groups Following Propensity Score Matching** | | | | |
| --- | --- | --- | --- | --- |
|  | N (%) or Mean (Standard Deviation) | | Standardized Differences | P-value |
| Characteristic^†^ | Transitions Clinic Network (n=94) | Matched Comparison Group (n=94) |  |  |
| Release year |  |  | 0.08 | 0.259 |
| 2012 | 1 (1.1) | 0 (0) |  |  |
| 2013 | 29 (30.1) | 23 (24.5) |  |  |
| 2014 | 61 (64.9) | 68 (72.3) |  |  |
| 2015 | 3(3.2) | 3 (3.2) |  |  |
|  |  |  |  |  |
| Male | 76 (80.9) | 75 (79.8) | 0.01 | 0.855 |
|  |  |  |  |  |
| Race/ethnicity |  |  | 0.01 | 0.914 |
| White | 29 (30.9) | 31 (33.0) |  |  |
| Black | 51 (54.3) | 48 (51.1) |  |  |
| Hispanic | 14 (14.9) | 15 (16.0) |  |  |
|  |  |  |  |  |
| Age (at release) | 42.6 (±10.4) | 40.5 (±10.8) | 0.10 | 0.179 |
|  |  |  |  |  |
| Department of Corrections assessment score |  |  |  |  |
| Mental health | 2.0 (±0.8) | 2.1 (±0.9) | 0.03 | 0.738 |
| Substance abuse | 3.4 (±1.2) | 3.4 (±1.2) | 0.00 | 1.00 |
| Education | 2.4 (±0.8) | 2.7 (±0.9) | 0.14 | 0.051 |
| Vocation | 3.2 (±0.7) | 3.2 (±0.8) | 0.01 | 0.923 |
| Crime severity | 2.4 (±1.1) | 2.6 (±1.2) | 0.07 | 0.372 |
| Medical problems | 2.5 (±0.8) | 2.4 (±1.0) | 0.02 | 0.775 |
|  |  |  |  |  |
| Length of Last Incarceration (days) | 239.9 (±312.4) | 306.3 (±1109.9) ^‡^ | 0.04 | 0.579 |
|  |  |  |  |  |
| Type of Release |  |  |  |  |
| Under supervision (half way house, transitional supervision, and parole) | 38 (40.9) | 34 (36.2) | 0.05 | 0.512 |
| Other (end of sentence, other discharge) | 55 (59.1) | 60 (63.3) | 0.07 | 0.399 |
|  |  |  |  |  |
| Proportion of life spent in Department of Corrections | 41.9 (±20.0) | 41.6 (±19.2) | 0.01 | 0.904 |
| Behavioral health history |  |  |  |  |
| Any inpatient stay for mental health condition | 13 (13.8) | 11 (11.7) | 0.03 | 0.664 |
| Proportion life involved with Department of Mental Health and Addiction Services | 17.4 (±14.6) | 18.0 (±12.2) | 0.02 | 0.764 |
|  |  |  |  |  |
| Medical chronic condition |  |  |  |  |
| Myocardial infarction | 5 (5.3) | 4 (4.3) | 0.03 | 0.734 |
| Dementia | 3 (3.2) | 1 (1.1) | 0.08 | 0.315 |
| Diabetes without complications | 19 (20.2) | 15 (16.0) | 0.06 | 0.734 |
| Moderate or severe liver disease | 5 (5.3) | 4 (4.3) | 0.03 | 0.734 |
| Other gastrointestinal conditions | 51 (54.4) | 53 (56.4) | 0.03 | 0.771 |
| Opioid use disorder | 36 (38.3) | 37 (39.4) | 0.01 | 0.882 |
| Alcohol use disorder | 50 (53.2) | 50 (53.2) | 0.00 | 1.00 |
| Weighted Charlson Comorbidity Index score | 2.0 (±3.0) | 2.5 (±3.4) | 0.08 | 0.302 |
| Total number of chronic conditions | 1.1 (±1.6) | 1.3 (±1.7) | 0.05 | 0.556 |

^†^ Following matching, all participant characteristics were balanced, meaning there were no significant differences between the two groups.

^‡^ The large standard deviation of the matched comparison group (1109.9 days) is driven by one individual who was last incarcerated for over 10,000 days. Excluding this individual, the mean for the matched comparison group is 197.5 days and the standard deviation is 348.0 days.

| **Table 2: Average Monthly Criminal Justice System, Medicaid, and Total Costs per Individual By Group, Excluding Dually Eligible (Medicaid/Medicare) Participants** | | | | | | | |
| --- | --- | --- | --- | --- | --- | --- | --- |
|  | Transitions Clinic Network (n=90) | |  | Comparison Group (n=90) | | Mean in Differences (95% CI) | Significance^†^ |
| Costs | Mean | Standard Deviation |  | Mean | Standard Deviation |  |  |
| Criminal Justice System | $753 | $1,109 |  | $1,307 | $1,757 | -554 (-978, -125) | 0.012 |
| Medicaid | $1,801 | $3,512 |  | $1,374 | $2,583 | 427 (-492, 1357) | 0.354 |
| Total | $2,553 | $3,663 |  | $2,681 | $2,830 | 487 (-1146, 905) | 0.795 |

^†^ Significance is based on bootstrap results of 1000 samples.

| **Table 3: Average Monthly Probation Costs per Individual By Group Including Additional Probation Days** | | | | | | | |
| --- | --- | --- | --- | --- | --- | --- | --- |
|  | Transitions Clinic Network (n=94) | |  | Comparison Group (n=94) | | Mean in Differences (95% CI) | Significance^†^ |
| Costs | Mean | Standard Deviation |  | Mean | Standard Deviation |  |  |
| Probation | $40 | $96 |  | $112 | $166 | 72 (33, 111) | <0.001 |

^†^ Significance is based on bootstrap results of 1000 samples.
